# New Volume and New Editorial Management

**Published:** 1853-06

**Authors:** 


*New volume and new editorial management.*—With the present No., the Buffalo Medical Journal enters on the ninth year of its existence. Having been the sole occupant of the tripod for the past eight years, we begin to find our position rather lonely, and, accordingly we have induced a medical friend to take a seat with us for the coming twelve months. The name of our associate appears on the title page. His participation in the labors of editorship will probably commence with the No. for the next month.

We trust this new arrangement will prove satisfactory to all parties. How much enjoyment Dr. Hunt may derive from it he will be able to state after a fair trial. For ourselves, we anticipate something more substantial than the dignity of a senior editor, viz., the relief which may be expected from a division of labor. Our readers, we are sure, will reap an advantage from the assistance of our co-laborer, with whom they are already acquainted as the author of several able papers that have appeared in our columns over his own signature, and the *Smelfungus* papers.

Under the present arrangement, each editor will be responsible for his own articles, and not for those of his colleague. Contributions to the editorial department by the junior editor will be signed by the initials of his name. Articles by the senior editor will be without signature.

In concluding this notice we may be permitted to say that the present condition of this Journal is sufficiently prosperous to meet the views of its proprietors, and to secure its continuance. Its circulation has steadily increased, without much effort on the part of the publishers to bring it to the notice of the profession, and in the number of its patrons, as well as other manifestations of the favorable consideration in which it is held, those most interested in its progress can find no cause of complaint. We shall be excused for making this statement, inasmuch as we do not often obtrude private matters upon the attention of our readers, and, moreover, none can be more sensible than ourselves of the fact that the success of the work is mainly due to the numerous friends who have sustained it by their valuable communications.

